# Prevalence in Britain of abnormal prion protein in human appendices before and after exposure to the cattle BSE epizootic

**DOI:** 10.1007/s00401-020-02153-7

**Published:** 2020-03-30

**Authors:** O. Noel Gill, Yvonne Spencer, Angela Richard-Loendt, Carole Kelly, David Brown, Katy Sinka, Nick Andrews, Reza Dabaghian, Marion Simmons, Philip Edwards, Peter Bellerby, David J. Everest, Mark McCall, Linda M. McCardle, Jacqueline Linehan, Simon Mead, David A. Hilton, James W. Ironside, Sebastian Brandner

**Affiliations:** 1 STI and HIV Department and CJD Section’ Blood Safety, Hepatitis, STIs and HIV Division Public Health England National Infection Service, 61 Colindale Avenue, London, NW9 5EQ United Kingdom; 2 Pathology and Animal Sciences Department Science Directorate Animal and Plant Health Agency Addlestone, Surrey, KT15 3NB United Kingdom; 3 Department of Neurodegenerative Disease, UCL Queen Square Institute of Neurology Queen Square, London, WC1N 3BG United Kingdom; 4 Division of Neuropathology, The National Hospital for Neurology and Neurosurgery, University College London Hospitals NHS Foundation Trust Queen Square, London, WC1N 3BG United Kingdom; 5 Virus Reference Department Public, Health England National Infection Service, 61 Colindale Avenue, London, NW9 5HT United Kingdom; 6 Department of Cellular and Anatomical, Pathology University Hospitals Plymouth, Plymouth, PL6 8DH United Kingdom; 7 National Creutzfeldt-Jakob Disease Research and Surveillance Unit Centre for Clinical Brain Sciences, University of Edinburgh, Western General Hospital, Edinburgh, EH4 2XU United Kingdom; 8 MRC Prion Unit at UCL, UCL Institute of Prion Diseases Courtauld Building, 33 Cleveland Street, London, W1W 7FF United Kingdom

**Keywords:** Bovine spongiform encephalopathy, BSE, Variant CJD, vCJD, Surveillance, Subclinical infection, Prion protein, PrP, Prion disease, Transmissible proteinopathies, Appendix, Tonsil, Lymphoreticular tissue

## Abstract

Widespread dietary exposure of the population of Britain to bovine spongiform encephalopathy (BSE) prions in the 1980s and 1990s led to the emergence of variant Creutzfeldt-Jakob Disease (vCJD) in humans. Two previous appendectomy sample surveys (Appendix-1 and -2) estimated the prevalence of abnormal prion protein (PrP) in the British population exposed to BSE to be 237 per million and 493 per million, respectively. The Appendix-3 survey was recommended to measure the prevalence of abnormal PrP in population groups thought to have been unexposed to BSE. Immunohistochemistry for abnormal PrP was performed on 29,516 samples from appendices removed between 1962 and 1979 from persons born between 1891 through 1965, and from those born after 1996 that had been operated on from 2000 through 2014. Seven appendices were positive for abnormal PrP, of which two were from the pre-BSE-exposure era and five from the post BSE-exposure period. None of the seven positive samples were from appendices removed before 1977, or in patients born after 2000 and none came from individuals diagnosed with vCJD. There was no statistical difference in the prevalence of abnormal PrP across birth and exposure cohorts. Two interpretations are possible. Either there is a low background prevalence of abnormal PrP in human lymphoid tissues that may not progress to vCJD. Alternatively, all positive specimens are attributable to BSE exposure, a finding that would necessitate human exposure having begun in the late 1970s and continuing through the late 1990s.

**Electronic supplementary material:**

The online version of this article (10.1007/s00401-020-02153-7) contains supplementary material, which is available to authorized users.

## Introduction

The dietary exposure of the population of Britain to bovine spongiform encephalopathy (BSE) prions in the late 1980s and early 1990s [45] led to the emergence of variant Creutzfeldt-Jakob Disease (vCJD) [48]. This form of prion disease is characterised by a strain that is different from other forms of human prion disease [20], giving rise to a distinct clinical picture, biochemical pattern [9] and histopathological appearance [48]. People affected by vCJD were significantly younger than those succumbing to sporadic forms of prion disease [47]. A highly characteristic feature of vCJD is the accumulation of abnormal prion protein (abnormal PrP, or also designated PrP^Sc^), a misfolded form of the normal host prion protein (PrP^C^) [2] in the lymphoreticular system, such as lymph nodes, tonsils, spleen and lymphoid follicles in intestinal organs [19, 21, 24], something that is absent in sporadic CJD [19, 21], or other transmitted forms such as kuru [6, 10] or iatrogenic CJD [18]. The presence of abnormal PrP in lymphoreticular tissues precedes involvement of the central nervous system (CNS) [5, 22] and it was inferred that the prevalence of vCJD carrier status in the population could be estimated through testing appendix and tonsil specimens removed at elective operations.

Whilst the number of clinical vCJD cases so far identified is relatively small, at 178 in the United Kingdom, it is conceivable that a relatively large number of people are infected. A particular public health concern is that infected individuals might pass the infection to others through surgical instruments, blood donation, or tissue and organ donations. This could lead to a self-sustaining secondary epidemic of vCJD in the population [16]. Several expensive and ongoing measures are in place to mitigate these risks [1, 37, 42]. A number of studies have been conducted to improve the accuracy of vCJD abnormal PrP prevalence estimates [7, 11, 15].

The first study of appendix and some tonsil tissue (the Appendix-1 Study), from operations conducted between 1995 and 1999, found three positive samples out of 12,674 screened for abnormal PrP using the immunohistochemistry (IHC) technique [23], Supplementary Table 1, online resource. This equates to a prevalence of 237 per million overall (95% confidence interval (CI) 49–692 per million) or one per 4,000 of the British population in the 1961 to 1985 birth cohort, the cohort in which most vCJD cases have arisen.

The second and larger Appendix-2 IHC screening study comprised appendix samples from operations conducted between 2000 and 2012. Sixteen abnormal PrP prion-positive samples were found in 32,441 appendix samples from those born between 1941 and 1985 (Supplementary Tables 1, 2, online resource), a prevalence of 493 per million (95% CI 269–1596 per million), or one in 2000, of the British population [17]. During completion of Appendix-2, the Transmissible Spongiform Encephalopathies (TSE) Risk Assessment Subgroup of the Advisory Committee on Dangerous Pathogens (ACDP TSE Risk Subgroup), the successor to the SEAC (Spongiform Encephalopathy Advisory Committee), advised that a further similar survey should be conducted on tissues from a ‘control’ population, i.e. one thought to have been unexposed to BSE.

A third national survey (the Appendix-3 Study) was therefore designed to test the hypothesis that there would be an absence of samples positive for abnormal PrP in appendices removed from people from outside the population considered most at-risk of acquiring vCJD from BSE via the food chain, i.e. appendices collected from operations performed either before 1980 (“historical”), or after 2000 in those born since 1996 (“new”). These periods and birth cohorts, in Britain, are composed of persons whose exposure to BSE prions through the food chain is expected to have been extremely low or negligible. The study was designed to be both feasible and sufficiently large to show a prevalence difference from Appendix-2 [17]. If there was a real prevalence difference, then the observed prevalence in the Appendix-3 survey would have needed to be zero or very low essentially no more than one positive from 15,000 or two positives from 20,000 appendix samples.

## Materials and methods

The sample collection, data handling and technical procedures, including equipment and antibodies, were performed as for the Appendix-2 survey [17]. However, the inclusion of historical samples dating from the 1960s required wax block re-embedding as described below.

### Consent and ethics

**Fig. 1:**
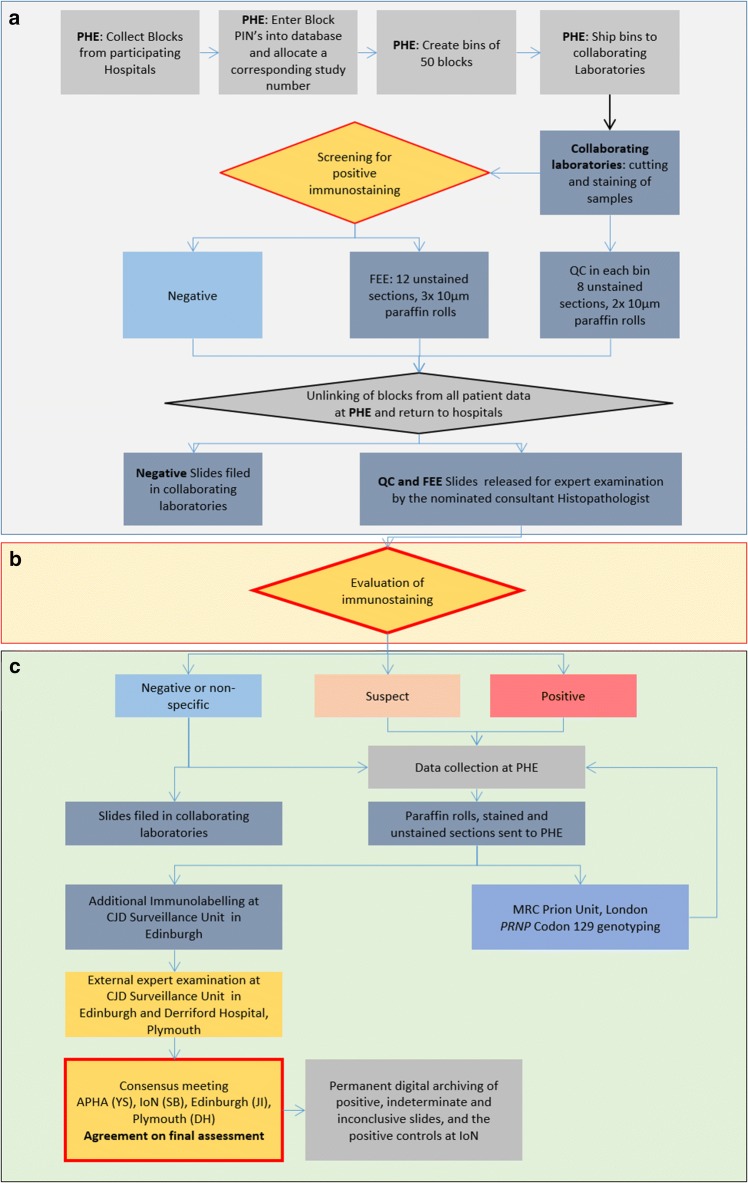
Flowchart to illustrate the pathway of tissue blocks from collection, testing and return. **a** Collection of blocks, database entry and allocation to bins of 50 blocks at PHE. Blocks were then sent to the participating laboratories, where they were sectioned and stained. Blocks with positive labelling were further sectioned (12 sections, and 3 × 10 μm paraffin rolls), but returned to PHE for unlinking prior to expert examination. **b** Following unlinking, “FEE sections” were examined by the histopathologists. **c** Confirmation of a “negative, non-specific, suspect, or positive” sample, as defined in the Methods section. The result was communicated to PHE and suspect or positive as well as unstained slides were sent to the external experts (JI, Edinburgh and then DH, Plymouth). Additional immunohistochemical preparations were prepared in the laboratory in Edinburgh when necessary. Consensus meetings were held to agree on a final assessment and slides were digitally archived with whole slide imaging

The unlinked anonymous methodology used in this study required all specimens to be irreversibly anonymised before any expert examination began (Fig. 1). The design ensured there was no possibility of tracing the identity of any individual from whom ‘positive’ tissue originated, either directly or indirectly, therefore patient consent was not required [25]. The study design received a favourable ethical opinion from Trent Research Ethics Committee (REC reference number: 08/H0405/69).

**Table 1:**
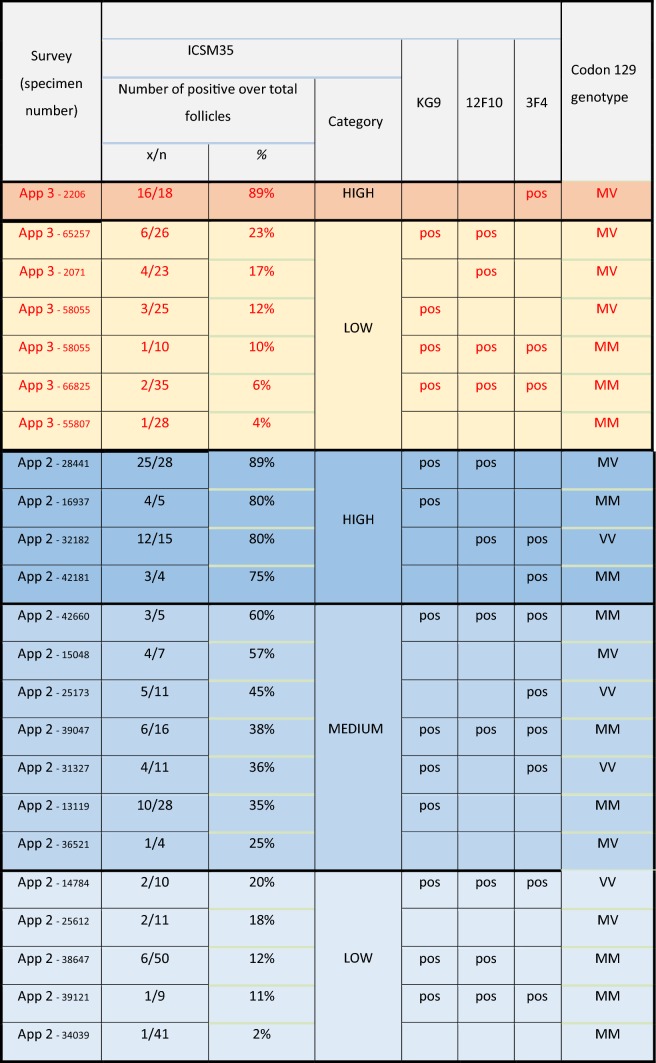
Immunoreactivity and *PRNP* Codon 129 genotype: comparison between the two prevalence studies

Appendix-3 survey data is colour-coded in red hues with red letters and Appendix-2 in blue hues and black letters, with descending intensity corresponding to the staining intensity of the follicles). Left column: Survey (App-2 or App-3) and serial number of the sample. The following three columns list the results from the ICSM35 staining with percentage of positive follicles in each sample followed by a “category” of staining intensity. The next three columns indicate if these follicles were also positively labelled with the other antibodies, KG9, 12F10 and 3F4. The column on the right indicates the genotype on codon 129 of the *PRNP* gene

### Sample collection, anonymisation, and data handling

The study plan was to collect and archive sufficient (up to 40,000) appendix samples from participating pathology departments in Britain, of which 20,000 were from appendectomies that took place before 1980, while another 20,000 were from operations between 2000 and 2014 from those born in 1996 or later. It was anticipated that about 25% of the samples would not be suitable for IHC testing, so a sample size of approximately 30,000 would be examined for the presence of abnormal PrP.

The source appendix tissues, archived in histology wax blocks (formalin fixed, paraffin embedded (FFPE)) were collected from 44 hospitals across Britain. Participating hospitals (Supplementary Table 12, online resource) were visited by trained technical staff from Public Health England (PHE) and the Animal and Plant Health Agency (APHA) to retrieve samples for inclusion in the study, except where they requested to retrieve the samples themselves. Appendectomy specimens that met the inclusion criteria were identified by searching day books, requisition forms, spreadsheets and/or histopathology department databases. Biopsies were mainly coded using Systematized Nomenclature of Pathology (SNOP) or on the Systematized Nomenclature of Medicine (SNOMED) that classified tissue type and morphology as numerical codes, thus allowing identification of the target specimens and where they were stored. Single blocks from each case were selected and couriered in large batches to the PHE co-ordinating laboratory.

After application of a study number to each specimen (i.e. study-specific sample number), PHE forwarded the appendices in collections (“bins”) of 50 blocks to the two collaborating prion screening laboratories, the Department of Neurodegenerative Diseases at the UCL Queen Square Institute of Neurology (UCL-IoN) and the Pathology Department at the APHA laboratory at Weybridge. These laboratories performed sectioning, IHC staining, initial screening and expert microscopic examination. After quality assessment of the sectioning and IHC staining (see below), and initial screening, but before expert assessment of the sections, the source appendix samples were returned to PHE. Here, details that could identify or trace back to any individual patient were unlinked from the study number. Non-identifying details needed for analysis gender, 5-year birth cohort, and broad geographical area where the original appendectomy hospital was sited were retained. After this anonymisation step the expert examination of the slides commenced (see below and flowchart in Fig. 1).

### Preparation of sections and immunohistochemical detection of abnormal PrP

Five automated Ventana Discovery XT immunohistochemistry (IHC) instruments (Roche, Burgess Hill, UK) and identical protocols were used by the two screening laboratories. A primary set of three sections was cut from each appendix block. Blocks that were processed and mounted on wooden chucks were first re-embedded onto Tissue-Tek cassettes to enable microtomy with current equipment. Abnormal PrP was detected using mouse monoclonal anti-PrP antibodies KG9 (PrP epitopes aa140-180; Dilution 1:500, TSE Resource centre, Roslin Institute Edinburgh, UK) on one section and ICSM35 (aa93-102; Dilution 1:1000 of 100 µg/ml, D-Gen, UK) [4, 11, 15] on a second section and visualised using a peroxidase-diaminobenzidine (DAB) Detection Kit (DAB Map Ventana Medical System) [17]. A first microscopic assessment and screening was performed by a histology technician to determine the quality of the immunostaining and morphology of the section and to assess whether the originating block was of sufficient quality and interest to continue through to the secondary preparation stage. At this stage, specimens were classified as either ‘non-reactive’, ‘unsuitable’ or ‘for expert examination’ (FEE) (Fig. 1).

An ‘unsuitable’ sample was defined as lymphoid tissue containing fewer than five secondary lymphoid follicles, or other (non-appendix) tissue which may have been collected or retrieved in error. An ‘FEE’ sample was one in which the technician was unsure about what was seen, or which showed any evidence suggestive of staining of follicular dendritic cells (FDC).

The abnormal accumulation of PrP, i.e. staining detectable above the methodological threshold set by technique optimisation in control populations, is considered a surrogate for detection of abnormal PrP, and henceforth referred to as “(accumulation of) abnormal PrP’’. To investigate FEE specimens, secondary preparation included repeating the staining and/or applying alternative anti-PrP monoclonal antibodies (12F10, Cayman Chemical, UK and 3F4, Signet, UK). From every FEE block, 17 additional sections were prepared and mounted on glass slides for further immunostaining or archiving. In addition, an equivalent of ca. 30 μm of tissue (usually in two separate cuts) was distributed into three Eppendorf tubes (10 μm equivalent per tube) for potential future genotyping and other investigations including transmission studies.

Four of the 17 slides were immunostained with ICSM35, KG9, 12F10 and 3F4 using the same technical procedure as described above. Although ‘anonymised’ throughout the primary and secondary preparation stages, these slides were NOT examined for the presence of abnormal PrP until after the ‘unlinking’ stage was complete (Fig. 1).

### Expert examination

Expert examination at IoN (SB) or APHA (MMS/YS) categorised samples as either positive, suspect, non-specific or negative (Fig. 1b) [17]. A ‘positive’ sample had to show immunostaining of a characteristic FDC network within a germinative centre of a follicle and at least one follicle had to contain a small network of immunopositive FDCs. The positive FDCs had to be present either in the same follicle in consecutive sections, or in a different follicle on a deeper section. A ‘suspect’ sample would have weak staining in a follicle, but in an atypical pattern, or weak or equivocal staining not reproduced in consecutive sections. Specimens classified as ‘non-specific’ had non-specific staining, or diffuse reactivity of the entire follicular area, or poor definition of the positively labelled cells, or very weak immunoreactivity in all sections including the repeats. Samples were classed ‘negative’ when they showed no immunostaining or reactivity at all, or presented only non-specific staining of non- FDC structures within follicles (such as macrophages, non-specific (background) staining inside the follicles), or staining of structures outside the follicles such as nerve fibres, macrophages, mucosa epithelium and myofibroblasts, and occasionally small parasites. All section sets of interest (positive and suspect immunostaining) were then referred to other experts (JWI) for staining of spare sections at the National CJD Research and Surveillance Unit. Each expert prepared written reports on each specimen set that were returned to the co-ordinating centre without knowledge of the other experts’ conclusions on the same specimen set. Finally, all the expert histopathologists met, and together reviewed each written report and slide set to arrive at a consensus opinion of the findings (Fig. 1c).

### Determination of *PRNP* codon 129 genotype

The *PRNP* codon 129 genotype of positive samples, and a selection of others, was determined using allele discrimination with minor groove binding (MGB) probes [17]. For primary assay, an RT-PCR was used, which was confirmed with a PCR based restriction endonuclease analysis (Table 1).

### Slide management and archiving

After completion of expert examinations, all the stained, and any remaining unstained, slides were stored separately at both IoN and APHA. Once all testing was completed, all of the sets of slides, both stained and unstained, and the additional tissue sections, were sent to IoN and stored for any follow up investigations on samples that were deemed positive or suspect. Images of all slides assessed as positive or suspect were digitally archived with a LEICA SCN400 scanner (LEICA, Milton Keynes UK) at 40 × magnification (0.25 μm/pixel) and are stored on a file server hosted by UCL. Slides are managed with Leica Slidepath software.

## Results

**Fig. 2:**
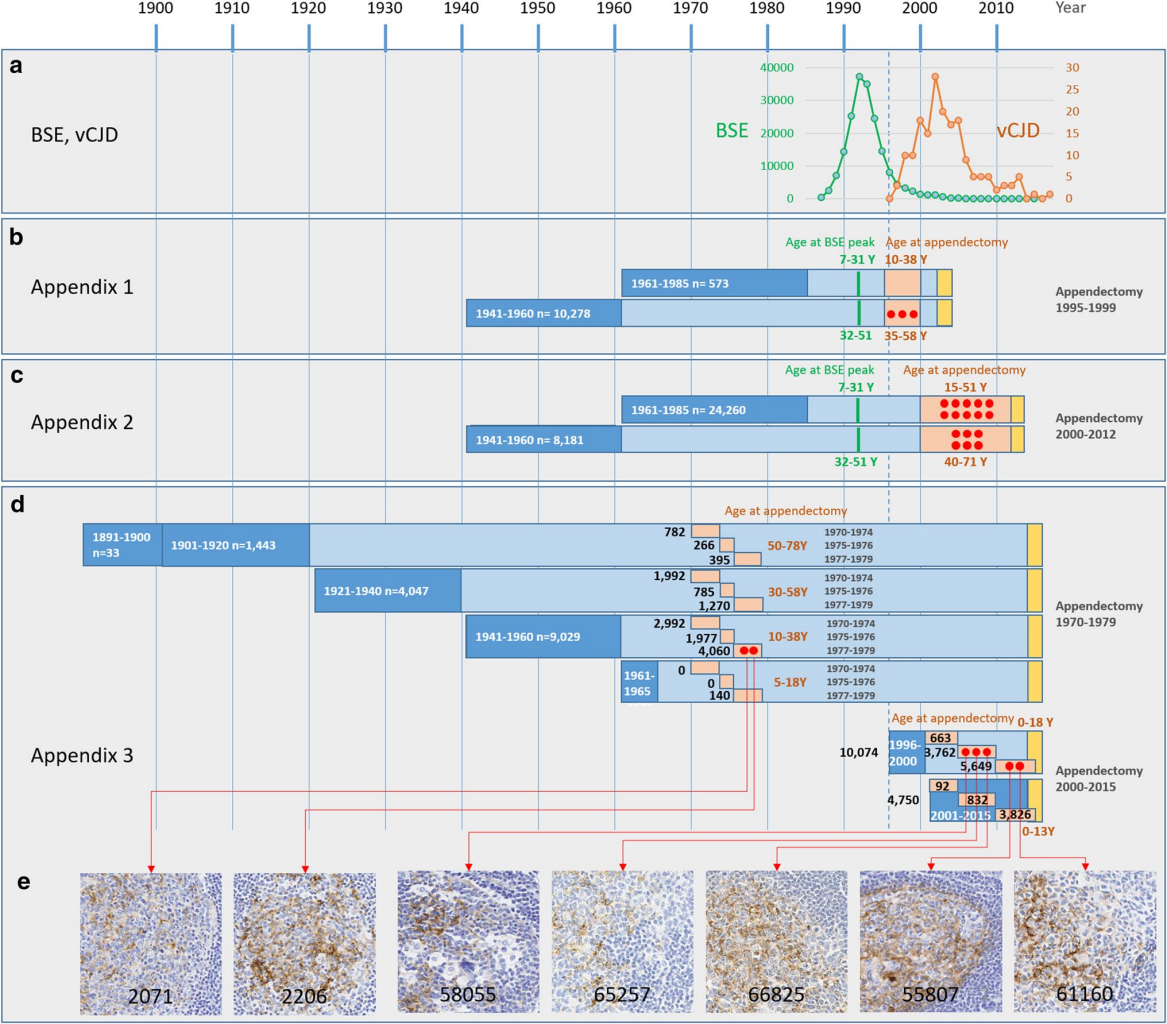
Outcome of previous, and the current, studies in relation to cases of BSE and vCJD. **a** Incidence of BSE (green) and vCJD (orange) in relation to the cohorts of the three studies. The dotted line indicates the introduction of the ban of bone meal supplement to cattle feed in 1996. **b** Cohorts of the Appendix-1 Study, with indication of the age at the BSE peak, and the age at appendectomy. Dark blue, birth years; orange, appendectomy years; yellow, study years). Red dots indicate positive samples in the cohort. **c** Appendix-2 Study with the same birth cohorts as in Appendix-1, with the same age at BSE peak, but higher age at appendectomy. 16 positive samples were identified across the two cohorts. **d** Current study with illustration of the birth cohorts, and the respective ages at the time of appendectomy. Two positive samples were identified in the 1970–1979 appendectomy cohort and five samples in the 2000–2015 appendectomy cohort. **e** Illustration of typical staining patterns representative for each positive sample (labelled with study number)

Abnormal PrP accumulation was detected within the FDCs of seven appendices out of 29,516 suitable samples examined. Two of the seven positive samples were from the 14,692 appendices removed at operations conducted in 1962 through 1979, and both these positive samples were from the 5,865 appendices removed in 1977 through 1979 (Fig. 2, Supplementary Tables 3, 5 and 6, online resource). The other five positive samples were found in the 14,824 appendices from people born in 1996 or later and removed at operation in 2000 through 2014: all five were in the sub-group of 10,074 born in 1996 through 2000. Therefore, none of the seven positive appendices were in specimens removed in 1976 or earlier, nor in patients born in 2001 or later (Fig. 2, Supplementary Table 4, online resource). In addition, using the available clinical data on operations involving the 178 known vCJD cases within the UK, it was deduced that none of the seven positive appendices could have been in tissue that originated from these known vCJD cases.

**Fig. 3:**
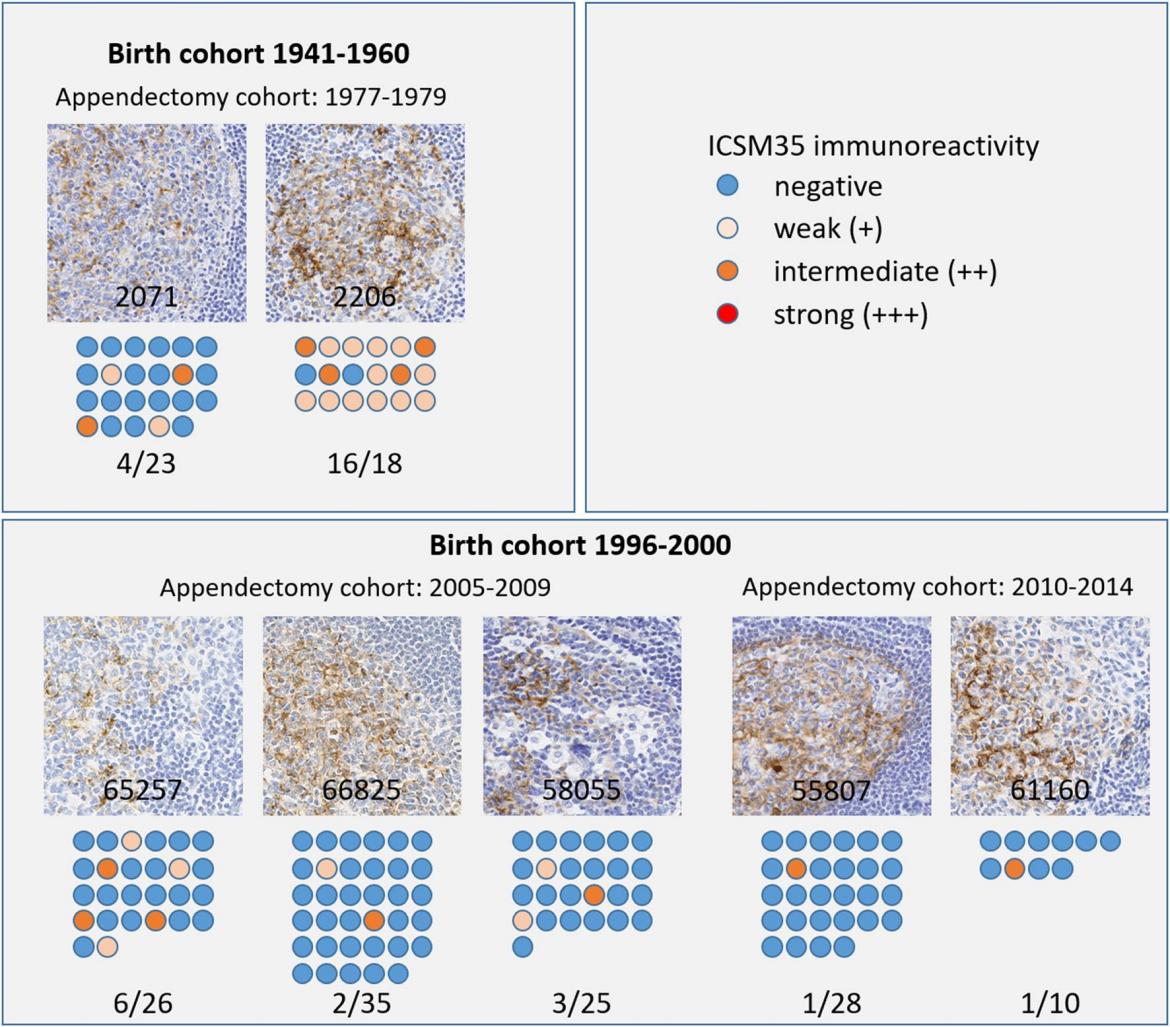
Illustration of all positive samples and indication of the ratio of positive follicles in each appendix sample. The colour codes indicate negative, weak, intermediate, or strong immunoreactivity with ICSM35 immunostaining. No morphological difference of staining patterns between the two birth cohorts

Whilst almost all patients with vCJD were homozygous for methionine at *PRNP* codon 129, in the previous Appendix-1 and Appendix-2 Studies, the valine allele was present in some of the positive appendix samples, and at a higher rate than expected for the UK population genotype frequency. In this Appendix-3 Study, four of the seven positive samples were codon 129 heterozygotes and two of these were from the cohort born in 1996 or later (Table 1). There was no discernible relationship between the genotypes of positive samples in either the Appendix-3 or -2 Studies  and the indicators of immunoreactivity ‘strength’ (proportion of follicles positive and number of antibodies showing positivity) (Table 1a, Figs. 2, 3). Moreover, critical appraisal of the histological findings in both studies showed no consistent differences between any of the positive samples.

The statistical analysis found no difference between the prevalence observed in the Appendix-2 Study of 493 per million (95% CI: 282 to 801 per million) and the Appendix-3 Study prevalence in appendices removed between 1962 through 1979 of 136 per million (95% CI: 16–492 per million; exact *p* = 0.08), nor with the Appendix-3 Study prevalence in appendices from those born in 1996 through 2000 of 337 per million (95% CI: 110–787 per million; exact *p* = 0.64). When the two groups (before 1980 and after 1996) in the Appendix-3 Study were combined, the central prevalence estimate for these groups was around 1 in 4,200 (240 per million), compared with the 1 in 2,000 (500 per million) from the Appendix-2 Study. However, this difference is not statistically significant. The results are also very similar to the Appendix-1 Study which examined samples from the 1961 through 1985 cohort and found three positive samples in roughly 12,000 tested, a rate of positivity of 1 in 4000 (250 per million).

Further post-hoc investigation of the year of removal of appendices in Appendix-3 showed that the samples that were positive came from the ‘latest’ taken of the pre-1980 appendices (both were from 1977–1979 removals, Fig. 2) or from the patients born ‘earliest’ in the cohort born from 1996 (all five were born 1996–2000, Fig. 2), so in both cases closer to the population previously considered at higher risk. Whilst this is an interesting observation, it should be noted that this clustering of cases closer to the higher risk populations was not statistically significant in this post-hoc assessment (*p* = 0.36 for taken 1970–1974 vs. 1975–1976 vs. 1977–1979, Supplementary Tables 7 and 8, online resource, and *p* = 0.18 for born 1996–2000 vs 2001–2015, Supplementary Tables 10 and 11, online resource).

## Discussion

The Appendix-3 Study was designed to measure the prevalence of abnormal PrP in appendices removed in operations performed before 1980 (historical), and after 2000 in those born since 1996 (new), i.e. in appendices taken from outside the population considered most at-risk of acquiring vCJD from BSE-related prions in the food chain. The overall prevalence of immunopositive samples found in these groups that were assumed to be unexposed to BSE was not lower than the prevalence in the most highly BSE-exposed cohort surveyed in the Appendix-2 Study. Examination of the available data on the appendectomy history of each human vCJD case to date showed that none of the positive appendices from this study (Appendix-3), nor the Appendix-2 Study [17], could have come from the 178 known vCJD cases in the UK.

The absence of a consistent difference between individual positive samples within Appendix-3 or between Appendix-2 and -3 is noteworthy. It might not have been possible to infer from these data alone that differences in immunostaining pattern of individual samples relate to source or strain, rather than host, or age of sample. Had we seen such differences it could have suggested different sources for the abnormal PrP detected.

One question is whether the IHC staining found in these prevalence studies was necessarily related to vCJD. The pattern of the staining observed in the positive specimens, however, is highly distinctive and consistent with that found in vCJD cases (both before and after onset of clinical symptoms). The abnormal accumulation of PrP in lymphoid tissue, as detected by immunohistochemistry, has only ever been found in humans with vCJD, and not in other human prion diseases such as sCJD [19, 20], and the transmitted forms of iatrogenic CJD [18] or Kuru [6, 10].

Two interpretations of the prevalence of abnormal PrP in different populations in Britain may be given. First, there is no significant difference in the prevalence of vCJD-related abnormal PrP between any of the appendix survey populations, i.e. there is a low background prevalence of abnormal PrP in human lymphoid tissues that may not progress to vCJD. This background prevalence is unrelated to the intensity and extent of dietary exposure to BSE. The alternative interpretation is that although there is no statistical difference in the prevalence of vCJD-related abnormal PrP across birth and exposure cohorts in the populations studied, the central estimates vary in a direction consistent with the changing intensity over time of the observed BSE epidemic in cattle. All positive specimens may therefore be attributable to BSE exposure.

This second interpretation, however, suggests that human exposure began in the late 1970s and continued through the late 1990s, albeit at a much lower rate than in the mid-1980s. Although cases of BSE were not described until 1986, back-calculation models indicate that cases could have been occurring, and infectivity possibly entering the food chain, for several years before the disease was identified in cattle [8, 46]. In addition, the origins of BSE have never been unequivocally established [40], so it could have been present at a very low prevalence for a long time prior to its amplification through the animal feed chain [46]. Cases of BSE continued to occur in animals born after the total feed ban put in place in the UK in July 1996, the reinforced feed ban in Ireland in October of the same year and the total feed ban in the rest of EU in 2001 [40], and such cases could provide one possible route of exposure for the vCJD cases identified in the post-1996 birth cohort.

Additionally, it has been demonstrated that sheep are susceptible to BSE [30, 44], and the disease can transmit between sheep under field conditions [31]. It has never been isolated from commercial sheep populations, but it has been observed in goats [43]. Sheep-passaged  BSE can demonstrate increased ‘virulence’ on subsequent inter-species transmission [38, 44], and can cause disease indistinguishable from vCJD in transgenic mouse models [32, 38]. A non-bovine route of exposure is therefore hypothetically possible.

Neither interpretation, on its own, is entirely satisfactory and it is possible to speculate about a combination of both. There could be ‘background’ prevalence in all groups plus some additional prevalence associated with BSE in the most highly exposed population. Detailed appraisal of the histological findings, however, showed no consistent differences between the positive samples that might have indicated two or more different sources. A large study of a population entirely unexposed to BSE prions would be necessary to determine whether a background prevalence exists, and such a study would pose additional challenges to those faced when implementing the Appendix-3 survey.

Whichever interpretation is preferred, the contrast between the prevalence of abnormal PrP and the number of clinical vCJD cases seen to date (mid-2020) strongly suggests that possibly none of those in whom abnormal PrP is detected through an ante-mortem lymphoid tissue survey will develop any symptoms of prion disease.

New research proposals have been sought that utilise some of the archived additional slides and cuts of formalin fixed tissues from each positive appendix [Department of Health Policy Research Programme—Research call on vCJD 2016: https://clahrcprojects.co.uk/news/department-health-policy-research-programme-invitation-applications] (accessed March 2020). In response, laboratory investigations are underway to elucidate the nature of the immunopositive samples. One approach is using in vitro conversion models to amplify the abnormal prion prior to conducting Western blotting analysis and transmission studies in mice (Green A; personal communication). Another is attempting discrimination of vCJD infected from uninfected fixed tissues through DNA methylation array “profiling” (Mead S; personal communication).

A variety of risk management measures remain in place to limit the risks of person-to-person transmission of prions by blood transfusion or by re-use of surgical instruments in the general population. Whichever way the Appendix-3 Study is interpreted, the prevalence range of prion infection remains a concern, and maintenance of the full range of precautionary measures is a judgement that would need to be balanced against the costs and benefits of these risk reduction measures [1, 37, 42]. More specifically, it is reasonable to assume that the highest prevalence of asymptomatic infection is in the cohort that had greatest exposure to BSE and which contains all known clinical cases of vCJD, the 1961 to 1985 birth cohort [36]. The findings of the Appendix-3 Study, however, challenge the assumption that a specific cut-off date defines a low-risk population, i.e. those born after 1996. Therefore, the difference between the interpretations of the Appendix-3 prevalence has practical implications for risk management.

The discovery that not only PrP but also other proteopathic seeds such as amyloid-β can be iatrogenically transmitted between humans has in the last few years received significant attention [33]. Whilst experimental transmission of amyloid-β had been demonstrated for some years [12, 13, 34], the observation of human transmission of amyloid-β through contaminated human growth hormone [28, 33, 41] has prompted additional studies examining the potential transmission through other human-derived products such as dura mater transplants [14, 35], intravascular embolization material [3], and through surgical instruments [29]. The transmissibility into susceptible animals of amyloid-β contained in human growth hormone preparations has provided further evidence of the historic role of this product in the development of cerebral amyloid angiopathy (CAA), a potentially lethal vascular disease, in affected individuals [26, 39]. These observational and experimental studies have put new emphasis on the necessity of adequate surveillance of relevant human diseases, sensitive detection of proteopathic seeds other than PrP [27] and their effective decontamination, for example on surgical instruments and medical devices.

In conclusion, the Appendix-3 Study has not produced a clear answer to the question of whether the presence of abnormal PrP, as detected by IHC, in the British population is limited to those exposed to the BSE epizootic. The results raise the possibility of abnormal prion exposure both before the presumed BSE epizootic and after 1996 when exposure to BSE-related prions in the food chain in Britain was considered “extremely low”.

## Electronic supplementary material

Below is the link to the electronic supplementary material.

Supplementary file1 (DOCX 53 kb)
