# Supplementary material for: Prevalence in Britain of abnormal prion protein in human appendices before and after exposure to the cattle BSE epizootic
Source: Acta Neuropathol. 2020 Mar 30;139(6):965–76. doi: 10.1007/s00401-020-02153-7 (PMC7244468; doi:10.1007/s00401-020-02153-7)
Supplement: Supplementary file 1 — Supplementary file1 (DOCX 53 kb) [file 401_2020_2153_MOESM1_ESM.docx]

**Prevalence in Britain of abnormal prion protein in human appendices before and after exposure to the cattle BSE epizootic**

**Supplementary data**

Results from the Appendix-3 Study are shown broken down by birth cohort and year of operation in supplementary tables 1-10. Positive samples are shown in brackets. From Table 5, one can see that the differences in prevalence of both Appendix-3 study cohorts compared with the Appendix-2 Study prevalence were not significant. The point estimates of prevalence were, however, lower in the Appendix-3 Study. The prevalence difference between Appendix-2 and 3 (historical) were 357 per million and (new) 156 per million but with wide 95% confidence intervals including zero.

|  | **Study** | | | | | |
| --- | --- | --- | --- | --- | --- | --- |
|  | **1** | **2** | **3** | **4** | **5** | **6** |
| **Genotype** | **[1]** | **[4]** | **App-2**  **Data [2]** | **App 3**  **Pre-1980** | **App3**  **1996+** | **App2+**  **App3** |
| **MM** | 511 (44.1%) | 2,225 (42.8%) | **8** | **-** | **3** | **11** |
| **VV** | 132 (11.4%) | 654 (12.6%) | **4** | **-** | **-** | **4** |
| **MV** | 515 (44.5%) | 2,318 (44.6%) | **4** | **2** | **2** | **8** |
|  | 1,158 (100%) | 5,197 (100%) | **16** | **2** | **5** | **23** |

## **Supplementary table 1:** Codon 129 Association with Positive Appendix Samples - Appendix-2 Study (n=32,441); Appendix-3 Study Pre1980 Operations (n=14,692); Appendix-3 Study Born 1996+ (n=14,824). The left column indicates the genotyped 1:129 of the PRNP gene; Column 1: DNA controls from the UK, Scottish blood donors and the Edinburgh/Belfast blood donor cohort; column 2: MRC Prion Unit UK blood donors/1958 birth cohort samples; column 3: appendix 2 study in which a more representative picture of the frequency of genotypes and the variation of immunolabelling in these positive appendixes was given, with 50% of the 16 positive samples being 129MM, 25% 129MV and 25% VV compared with 43%, 45%, and 13% in the general population respectively; Column 4, current study (appendix-3) pre-1980 birth cohort; column 5 current study with cohort 1996 and later; appendix 2 and current study combined.

|  | **Operation year cohorts** | |
| --- | --- | --- |
|  | **Appendix 1 [3]** | **Appendix 2 [2]** |
| **Birth cohort** | **1995 to 1999** | **2004 to 2012** |
| **1941 to 1945** | **573** | **1,420 (1)** |
| **1946 to 1950** |  | **1,918 (1)** |
| **1951 to 1955** |  | **2,124 (1)** |
| **1956 to 1960** |  | **2,719 (3)** |
| **1961 to 1965** | **10,278 (3)** | **3,438** |
| **1966 to 1970** |  | **4,090 (3)** |
| **1971 to 1975** |  | **4,408 (2)** |
| **1976 to 1980** |  | **5,184 (3)** |
| **1981 to 1985** |  | **7,140 (2)** |
| **Total** | **10,851 (3)** | **32,441 (16)** |

**Supplementary Table 2**: Suitable samples from previous studies: Appendix-1 (operation years 1995 to 1999) and Appendix-2 (operation years 2004 to 2012), by birth and operation year cohort – positives in brackets.

| **Birth cohort** | **Operation year cohorts** | | |  |
| --- | --- | --- | --- | --- |
|  | **Pre-1975** | **1975 to 1976** | **1977 to 1979** | **Total** |
| 1891 to 1900 | 33 | - | - | 33 |
| 1901 to 1920 | 782 | 266 | 395 | 1,443 |
| 1921 to 1940 | 1,992 | 785 | 1,270 | 4,047 |
| 1941 to 1960 | 2,992 | 1,977 | 4,060 (2) | 9,029 (2) |
| 1961 to 1965 | - | - | 140 | 140 |
| **Total** | **5,799** | **3,028** | **5,865 (2)** | **14,692 (2)** |

**Supplementary table 3:** Suitable Appendix-3 samples from operations performed before 1980, by birth and operation year cohort – number of positive samples in brackets**.**

| **Birth cohort** | **Operation year cohort** | | |  |
| --- | --- | --- | --- | --- |
|  | **2000 to 2004** | **2005 to 2009** | **2010 to 2014** | **Total** |
| 1996 to 2000 | 663 | 3,762 (3) | 5,649 (2) | 10,074 (5) |
| 2001 to 2015 | 92 | 832 | 3,826 | 4,750 |
| **Total** | **755** | **4,594 (3)** | **9,475 (2)** | **14,824 (5)** |

**Supplementary table 4:** Suitable Appendix-3 samples from persons born in 1996 or later, by birth and operation year cohort – positive samples in brackets.

| **Appendix-3 sub-set** | **Appendix-3 Prevalence (95% CI)** | **Appendix-2 [2] Prevalence (95% CI)** | **Difference (95% CI)** | **Exact *p*** |
| --- | --- | --- | --- | --- |
| **Operations before 1980 (historical)** | 136 (16-492), [2/14692] | 493 (282-801), [16/32441] | 357 (0-664)* | 0.08 |
| **Born after 1996 (new)** | 337 (110-787), [5/14824] | 493 (282-801), [16/32441] | 156 (-226-538) | 0.64 |

**Supplementary table 5**: Prevalence comparison – Appendix-3 study compared to Appendix-2 Study (rates per million). *bottom end set to zero because of discrepancy between non-significant exact *p*-value and 95% CI excluding zero

| **Appendix-3 sub-set** | **Appendix-3 Prevalence (95% CI)** | **Appendix-1 and 2 Prevalence (95% CI)** | **Difference (95% CI)** | **Exact *p*** |
| --- | --- | --- | --- | --- |
| **Operations before 1980 (historical)** | 136 (16-492), [2/14692] | 439 (264-685), [19/43292] | 303 (-30-576) | 0.13 |
| **Born after 1996 (new)** | 337 (110-787), [5/14824] | 439 (264-685), [19/43292] | 102 (-254-457) | 0.82 |

**Supplementary table 6:** Comparison of Appendix-3 prevalence with the combined prevalence from Appendix-1 [3] and Appendix-2 [2] Studies

| **Appendix-3 sub-set** | **Pre-1975** | **1975-76** | **1977-79** | **Exact *p*** |
| --- | --- | --- | --- | --- |
| **Operations before 1980 (historical)** | 0 (0-636), [0/5799] | 0 (0-1218), [0/3028] | 341 (41-1231), [2/5865] | 0.36 |

**Supplementary table 7**: Comparison of prevalence within Appendix-3 by year of operation (historical) subdivided as pre-1975, 1975-1976, 1977-1979

| **Appendix-3 sub-set** | **Born Pre-1941** | **Born Post-1941** | **Exact *p*** |
| --- | --- | --- | --- |
| **Operations before 1980 (historical)** | 0 (0-668), [0/5523] | 218 (26-788), [2/9169] | 0.53 |

**Supplementary table 8:** Appendix-3 comparison of prevalence by birth cohort (within historical) study as pre-1941 and post-1941.

| **Birth Cohort** | **Appendix-3 prevalence (95% CI)** | **Appendix-1 and 2 prevalence (95% CI)** | **Difference (95% CI)** | **Exact *p*** |
| --- | --- | --- | --- | --- |
| **1941 -1960** | 222 (27-800), [2/9029] | 685 (252-1491), [6/8754] | 464 (-1641092) | 0.17 |

**Supplementary Table 9:** Comparison of prevalence within the same birth cohort (1941-1960) between Appendix-3 sub-set (operations before 1980) and Appendix-1 and 2 Studies combined.

| **Birth Cohort** | **1996 to 2000** | **2001-2015** | **Exact *p*** |
| --- | --- | --- | --- |
| **Born after 1996 (new)** | 496 (161-1158), [5/10074] | 0 (0-776), [0/4750] | 0.18 |

**Supplementary Table 10:** Appendix-3 comparison of prevalence by birth cohort for those born after 1996.

| **Appendix-3**  **Prevalence**  **(95% CI)** | **Appendix-1 and 2 Prevalence (95% CI)** | **Difference (95% CI)** | **Exact *p*** |
| --- | --- | --- | --- |
| 237 (95-489), [7/29516] | 439 (264-685), [19/43292] | 202 (62466) | 0.17 |

**Supplementary Table 11:** Comparison of overall Appendix-3 prevalence with combined Appendix-1 and 2 prevalence.

| **Hospital** | **Postcode** | **NHS Trust** |
| --- | --- | --- |
| Addenbrooke's Hospital | CB2 0QQ | Cambridge University Hospitals NHS Foundation Trust |
| Broomfield Hospital | CM1 7ET | Mid Essex Hospital Services NHS Trust |
| Colchester General Hospital | CO4 5JL | East Suffolk and North Essex NHS Foundation Trust |
| Conquest Hospital | TN37 7RD | East Sussex Healthcare NHS Trust |
| County Hospital, Stafford | ST16 3SA | University Hospitals of North Midlands NHS Trust |
| Cumberland Infirmary | CA2 7HY | North Cumbria integrated care NHS Foundation Trust |
| Darent Valley Hospital | DA2 8DA | Dartford and Gravesham NHS Trust |
| Darlington Memorial Hospital | DL3 6HX | County Durham and Darlington NHS Foundation Trust. |
| Derriford Hospital | PL6 8DH | University Hospitals Plymouth NHS Trust |
| Dorset County Hospital | DT1 2JY | Dorset County Hospital NHS Foundation Trust |
| East Surrey Hospital | RH1 5RH | Surrey and Sussex healthcare NHS Trust |
| Epsom General Hospital | KT18 7EG | Epsom and St Helier University Hospitals NHS Trust |
| Frenchay Hospital | BS16 1LE | Closed in 2014, now Southmead Hospitals |
| Friarage Hospital | DL6 1JG | South Tees Hospital NHS Foundation Trust |
| Hillingdon Hospital | UB8 3NN | Hillingdon Hospitals NHS Foundation Trust |
| James Cook University Hospital | TS4 3BW | South Tees Hospitals NHS Foundation Trust |
| John Radcliffe Hospital | OX3 9DU | Oxford University Hospitals NHS Foundation Trust |
| Maidstone Hospital | ME16 9QQ | Maidstone and Tunbridge Wells NHS Trust |
| Manchester Royal Infirmary | M13 9WL | Manchester University NHS Foundation Trust |
| Milton Keynes University Hospital | MK6 5LD | Milton Keynes University Hospital NHS Foundation Trust |
| Musgrove Park Hospital | TA1 5DA | Taunton and Somerset NHS Foundation Trust |
| New Cross Hospital | WV10 0QP | Royal Wolverhampton NHS Trust. |
| Nottingham City Hospital | NG5 1PB | Nottingham University Hospitals NHS Trust |
| Pinderfields Hospital | WF1 4DG. | Mid Yorkshire Hospitals NHS Trust |
| Queen Alexandra Hospital, Portsmouth | PO6 3LY | Portsmouth Hospitals NHS Trust |
| Queen Elizabeth II Hospital | AL7 4HQ | East and North Hertfordshire NHS Trust |
| Queen's Hospital, Burton | DE13 0RB | University Hospitals of Derby and Burton NHS Foundation Trust |
| Royal Bolton Hospital | BL4 0JR | Bolton NHS Foundation Trust |
| Royal Cornwall Hospital | TR1 3LQ | Royal Cornwall Hospitals NHS Trust |
| Royal Derby Hospital | DE22 3NE | University Hospitals of Derby and Burton |
| Royal Devon and Exeter Hospital | EX2 5DW | Royal Devon and Exeter NHS Foundation Trust |
| Royal Lancaster Infirmary | LA1 4RP | University Hospitals of Morecambe Bay NHS Foundation Trust |
| Royal Shrewsbury Hospital | SY3 8XQ | Shrewsbury and Telford Hospitals NHS Trust |
| Royal Sussex County Hospital | BN2 5BE | Brighton and Sussex University Hospitals NHS Trust |
| Salford Royal Hospital | M6 8HD | Salford Royal NHS Foundation Trust |
| Salisbury District Hospital | SP2 8BJ | Salisbury NHS Foundation Trust |
| Southampton General Hospital | SO16 6YD | University Hospital Southampton NHS Foundation Trust |
| St Thomas' Hospital | SE1 7EH | Guy's and St Thomas NHS Foundation Trust |
| Stoke Mandeville Hospital | HP21 8AL | Buckinghamshire healthcare NHS Trust |
| Sunderland Royal Hospital | SR4 7TP | South Tyneside and Sunderland NHS Foundation Trust |
| The Queen's Medical Centre, Nottingham | NG7 2UH | Nottingham University Hospitals NHS Trust |
| Torbay Hospital | TQ2 7AA | Torbay and South Devon NHS Foundation Trust |
| University Hospital of North Durham | DH1 5TW | County Durham and Darlington NHS Foundation Trust |
| Whipps Cross University Hospital | E11 1NR | Barts Health NHS Trust |
| Worcestershire Royal Hospital | WR5 1DD | Worcestershire Acute Hospitals NHS Trust |
| Wycombe Hospital | HP11 2TT | Buckinghamshire Healthcare NHS Trust |
| Yeovil District Hospital | BA21 4AT | Yeovil District Hospital NHS Foundation Trust |

**Supplementary table 12:** Participating hospitals

**Supplementary methods**

Once specimens were sorted ready for return to their correct histopathology department, the relevant data in the Administrative Database was re-configured into the following fields: birth cohort of patient at time of operation (worked out using the year of birth), year of operation group (i.e. 1970 to 1976 or 1977 to 1979), gender, broad geographical area of country and unique study number. This ‘collapsed’ data was transferred into the Survey Database. Data including the specimen number, hospital and a tick box of whether the specimen has been re-archived was also entered into the Specimen Return Database so that the survey team could keep track of the whereabouts of each specimen. The barcode labels with the unique study numbers were taken off the blocks before returning them to their original hospital. At this point, permission for expert examination was granted to the collaborating laboratory by PHE, as the limited demographic data associated with the unique study number of each block could not be used to link back to the original specimen number (see Figure 1). IoN and APHA did not have access to any patient identifying information. They were also not given permission to begin their expert examination on samples until all the relevant associated data had been irreversibly unlinked.

**References**

1 Bishop MT, Pennington C, Heath CA, Will RG, Knight RS (2009) PRNP variation in UK sporadic and variant Creutzfeldt Jakob disease highlights genetic risk factors and a novel non-synonymous polymorphism. BMC Med Genet 10: 146 Doi 10.1186/1471-2350-10-146

2 Gill ON, Spencer Y, Richard-Loendt A, Kelly C, Dabaghian R, Boyes L, Linehan J, Simmons M, Webb P, Bellerby P et al (2013) Prevalent abnormal prion protein in human appendixes after bovine spongiform encephalopathy epizootic: large scale survey. BMJ 347: f5675 Doi 10.1136/bmj.f5675

3 Hilton DA, Ghani AC, Conyers L, Edwards P, McCardle L, Ritchie D, Penney M, Hegazy D, Ironside JW (2004) Prevalence of lymphoreticular prion protein accumulation in UK tissue samples. J Pathol 203: 733-739 Doi 10.1002/path.1580

4 Mead S, Poulter M, Uphill J, Beck J, Whitfield J, Webb TE, Campbell T, Adamson G, Deriziotis P, Tabrizi SJ et al (2009) Genetic risk factors for variant Creutzfeldt-Jakob disease: a genome-wide association study. Lancet Neurol 8: 57-66 Doi 10.1016/S1474-4422(08)70265-5
